# Five new species of *Impatiens* (Balsaminaceae) from southern Western Ghats, Kerala

**DOI:** 10.3897/phytokeys.276.200226

**Published:** 2026-06-23

**Authors:** Sindhu Arya, Thomas Salvy, Thomas Manuel, Thomas Plammoottil Rogimon

**Affiliations:** 1 Division of Plant Taxonomy, Travancore Institute for Bioscience Research, Trivandrum, Kerala – 695024, India Division of Plant Taxonomy, Travancore Institute for Bioscience Research Trivandrum India; 2 Department of Botany, St. Berchmans College (Autonomous), Changanassery, Kerala –686101, India Department of Botany, St. Berchmans College (Autonomous) Changanassery India; 3 Karimpanackel House, Neeloor P.O., Kottayam, Kerala- 686651, India Unaffiliated Kottayam India; 4 Department of Botany, CMS College Kottayam (Autonomous), Kottayam, Kerala – 686001, India Department of Botany, CMS College Kottayam (Autonomous) Kottayam India

**Keywords:** Ernakulam, Idukki, *
Impatiens
*, new species, Western Ghats

## Abstract

Five new species of *Impatiens*, viz. *Impatiens flavispicata*, *I. filcyii*, *I. ninanii*, *I. xanthopetala*, and *I. berchmansiensis*, are described from the southern Western Ghats of Kerala. *I. flavispicata* resembles *I. courtallensis* and *I. herbicola*; *I. filcyii* is similar to *I. periyarensis* and *I. aliciae*; *I. ninanii* is allied to *I. cheruthoniensis*; *I. xanthopetala* shows similarity to *I. flavescens* and *I. inconspicua* and *I. berchmansiensis* is similar to *I. herbicola*. The newly described taxa are readily distinguished from their allied species by strong character combinations. Detailed descriptions, along with SEM analysis of pollen, photographs, and distribution maps, are provided.

## Introduction

*Impatiens* is a large, diverse group of annual and perennial herbs widely distributed across tropical and subtropical regions of the Old World and also in northern temperate regions (Stevens 2001; Yuan et al. 2004; Fischer 2004; Mabberley 2008). *Impatiens* is found in five distinct hotspots of diversity: tropical Africa, Madagascar, southern India and Sri Lanka, the eastern Himalayas, and Southeast Asia (Song et al. 2003; Janssens et al. 2019; Fischer et al. 2020; Raskoti and Ale 2022). Dessai and Janarthanam (2011) noted the presence of 210 species in India and also recorded high endemism across the Western Ghats and Himalayas. An array of floristic studies and inventories of the genus *Impatiens* has been widely discussed (Gamble 1915; Fyson 1932). There are 26 species and two varieties from the northern and central Western Ghats alone (Kumar and Sindhu 2024). The Western Ghats host 106 species of *Impatiens* with high endemism that corresponds to its adaptability in highly specific microhabitats (Bhaskar 2012). New species are regularly being reported from this region, viz. *I. saulierea* and *I. josephia* (Mani et al. 2018); *I. dindigulensis* (Ramasubbu et al. 2020); *I. achudanandanii*, *I. danii*, and *I. shailajae* (Arya et al. 2021); *I. godfreyi* (Richard et al. 2022); *I. minnamparaensis* (Sindhu et al. 2024); and *I. linnaei* (Satheshkumar et al. 2025).

Field exploration, carried out in the southern Western Ghats of the Kerala region, revealed several morphologically unique specimens of *Impatiens*. On the basis of critical evaluation of collected specimens, comparison with various herbaria, and literature review, these specimens were found to be distinct from all other known species. Hence, five of them are proposed here as species new to science.

## Materials and methods

Field surveys were carried out from July 2023 to March 2026. The plants were collected and submitted to the herbaria of CMS and RHK. The specimens collected from various regions were used for morphological comparison and were examined with specimens in the herbaria E, MH, and TBGT (acronyms according to Thiers 2026+). Photographs of habitat and habit were taken with a 1500D DSLR camera (Canon). Photographic images of floral parts were taken with a stereomicroscope (Zeiss, Germany). The terminology of morphological characters were in accordance with Grey-Willson (1980). Conservation assessment was conducted using the IUCN Red List categories and criteria (IUCN 2024). Analysis of relevant literature (Dessai and Janarthanam 2011; Bhaskar 2012; Hareesh et al. 2015; Prabhukumar et al. 2015, 2022; Ramasubbu et al. 2015, 2017; Singh 2016; Bhaskar and Sringeswara 2017; Mani et al. 2018; Arya et al. 2021; Biju et al. 2022; Mohan et al. 2021; Kumar and Sindhu 2024) and careful examination of preserved specimens at various herbaria (TBGT, MH, K, USF, and CALI) (acronyms according to Thiers 2026, continuously updated) were undertaken to complete the study. Furthermore, the distribution map was created using ArcGIS 10.8 (Fig. 8).

## Results and discussion

### Impatiens flavispicata

Taxon classificationPlantaeEricalesBalsaminaceae

Sindhu Arya, Salvy, Manuel & Rogimon sp. nov.

7E36F7B0-ECF6-56D8-9C1A-96D4E11729D0

urn:lsid:ipni.org:names:77382024-1

Fig. 1

#### Type.

India. Kerala, Ernakulam district, Mamalakkandam, along the streams of highland, 10.0090, 76.8076, 920 m a.s.l, 20 August 2023, *Sindhu Arya, Salvy, Manuel & Rogimon., 3010* (holotype CMS!, isotype RHK!).

**Figure 1.**
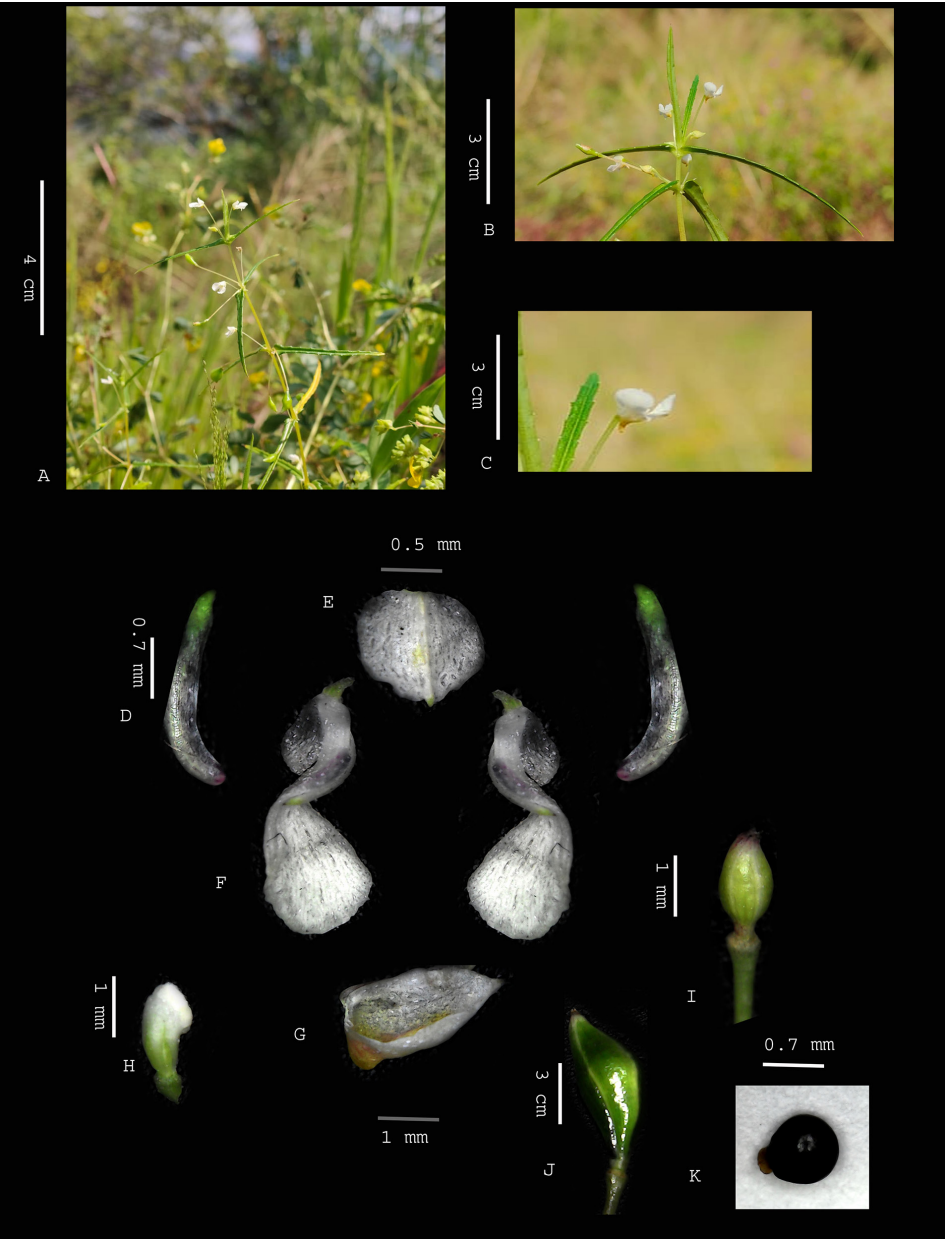
*Impatiens flavispicata*. **A**. Habit; **B**. Single plant; **C**. Flower, close-up; **D**. Lateral sepal; **E**. Dorsal petal; **F**. Lateral united petal; **G**. Lower sepal; **H**. Stamen; **I**. Gynoecium; **J**. Fruit; **K**. Seed.

#### Diagnosis.

*Impatiens flavispicata* is similar to *Impatiens courtallensis* Ramas. & Pandur. with respect to the presence of minute spur, opposite and decussate, lanceolate leaf and black color of the seed but distinct with respect shape of fruit and number of seeds (ovoid, 1–2 seeded in *I. flavispicata* vs. fusiform, 3–5 seeded in *I. courtallensis*), size of flower (4–5 mm vs. 1 cm), shape of dorsal petal (ovate, arched vs. orbicular, recurved), shape of lateral united petals (basal lobe minute, digitate, distal lobe obdeltoid vs. basal lobe oblong, distal lobe spherical), nature of seed (glabrous vs. caruncle with minute hairs), size of pollen grains (8 × 10 µm ovoid vs. 16 × 18 µm squarish).

The species is also distinct from its other similar taxon *I. herbicola* Hook. f with respect to size of the flowers (4–5 mm in *I. flavispicata* vs. 5–9 mm in *I. herbicola*) shape of the fruit (ovoid, 1–2 seed vs. gibbously ovoid, with many seeded), shape of lateral united petals (basal lobe digitate vs. oblong) shape of dorsal petal (ovate, faintly keeled vs. orbicular, thickly keeled) size and color of the pollen (8 × 10 µm, white vs. 21 × 23 µm, yellow).

#### Description (macromorphology).

Annual, erect herb, 18–20 cm high. ***Stem*** terete, branched, pubescent, extra nectarial structures with red dots; nodes prominent, slightly swollen, internode elongated, 2–2.3 cm. ***Leaves*** opposite and decussate, 3–3.8 × 0.8–1.0 cm long, shortly petiolate, petiole 0.4 mm, coriaceous, acuminate, entire, base truncate, slightly cordate, reflexed upwards, leaf margin distinctly serrate, leaf blade 3.2–4.1 cm, linear to lanceolate, extra petiolar glands present. ***Inflorescence*** 1–2 together from one axil. ***Flowers*** simple, pedicellate, axillary, 4–4.5 mm across, white with yellow spot on the throat. Pedicel 3–5 cm long. ***Lateral sepals*** two, linear-falcate, 0.5–1 mm long, not nerved, sparsely pubescent white, with yellow tip. ***Lower sepal*** cymbiform, tip of the lower sepal pointed, not recurved, 1.5–1.3 × 0.4–0.6 mm, horizontal, spur 0.3–0.5 mm, yellow. ***Petals*** dorsal ovate, arched 1.5–1.8 × 0.5–0.8 mm, beaked, dorsally keeled, keel faint, apiculate. ***Lateral united petals*** stipitate, not clawed, two lobed, basal lobe 1.1–1.5 mm minute digitate, margin smooth, distal lobe small, obdeltoid, dorsal auricle not prominent, end sharp. ***Anther*** white greenish 1–1.2 mm, pollen creamy white. ***Ovary*** ovoid, 0.5 mm long. ***Fruit*** capsule small, ovoid, turgid, 2.8–3.5 × 2–3 mm, acute, red shaded 1–2 seeded. ***Seed*** ovoid, smooth, glabrous, compressed, 1–2 × 0.5–1 mm, black.

#### Description (micromorphology).

***Pollen*** three colpate, ovoid, 8–10 µm × 10–11 µm. Surface pattern roughly reticulate forming a non-angular apocolpia which is prominent. Small hair-like structures are seen associated with the apocolpia. Mesocolpia is faintly distinguished. Muri measures ca. 2–2.5 µm, baculate, lumina ca. 1.8 µm, intra and inter-luminar bacules are present, 2–8 in each, often free or even fused (Fig. 7).

#### Phenology.

August to November.

#### Etymology.

The specific epithet flavispicata corresponds to the yellow-colored spur, which is diagnostic in this species.

#### Habitat and distribution.

*Impatiens flavispicata* is known only from the type locality at an elevation of 600–1,200 m a.s.l. It grows in open areas along rocky, steep landscapes with a limited number of individuals. Each plant spreads over an area of nearly 5–10 m^2^. The population shows luxuriant growth during the flowering season and is associated with *Utricularia caerulea* L., *Drosera burmannii* Vahl., and *Phyllocephalum keralense* Arya, Sindhu, Suresh, Sojan, Alen & V.S.A. Kumar.

#### Conservation status.

The new species is known from four subpopulations in the rocky terrain of Mamalakkandam, each separated by a distance of 400–500 m. The estimated extent of occurrence (EOO) is 5 km^2^, and the area of occupancy is less than 1 km^2^. The number of mature individuals in the area is expected to be 120–130. The locality is a major tourist spot; therefore, the possibility of population decline is high due to anthropogenic activity. Nevertheless, further study of the population is preferred before assigning any conservation status. Therefore, it is recommended that the species be categorized as Data Deficient (DD) based on IUCN (2024).

#### Other notes.

*Impatiens flavispicata* belongs to sect. *Uniflorae*, characterized by capsules that are short-fusiform, conspicuously turgid at the middle, ca. 1 cm long, an inflorescence that is a raceme with 1(–2) flowers, and ellipsoid seeds. Furthermore, the color of the flower, blotches on the throat, pollen surface, and shape of the spur are distinct characters that delineate *I. flavispicata* from other reported species.

#### Specimen examined (paratype).

*Impatiens flavispicata* India. Ernakulam district, Elamblassery, 10.0817°N, 76.8425°E, 550 m a.s.l., 14 September 2024, Sindhu Arya, Salvy, Manuel, & Rogimon; 3028 (RHK!).

### Impatiens filcyii

Taxon classificationPlantaeEricalesBalsaminaceae

Sindhu Arya, Manuel, Salvy & Rogimon sp. nov.

CD028736-DDB8-56D5-9226-4DFE5F5CA3F8

urn:lsid:ipni.org:names:77382025-1

Fig. 2

#### Type.

India. Kerala, Ernakulam district, Mamalakkandam, along the highland streams, 10.0968, 76.8154, 810 m a.s.l., 22 October 2023, *Sindhu Arya, Rogimon, Salvy & Manuel, 3045* (holotype CMS!, isotype RHK!).

**Figure 2.**
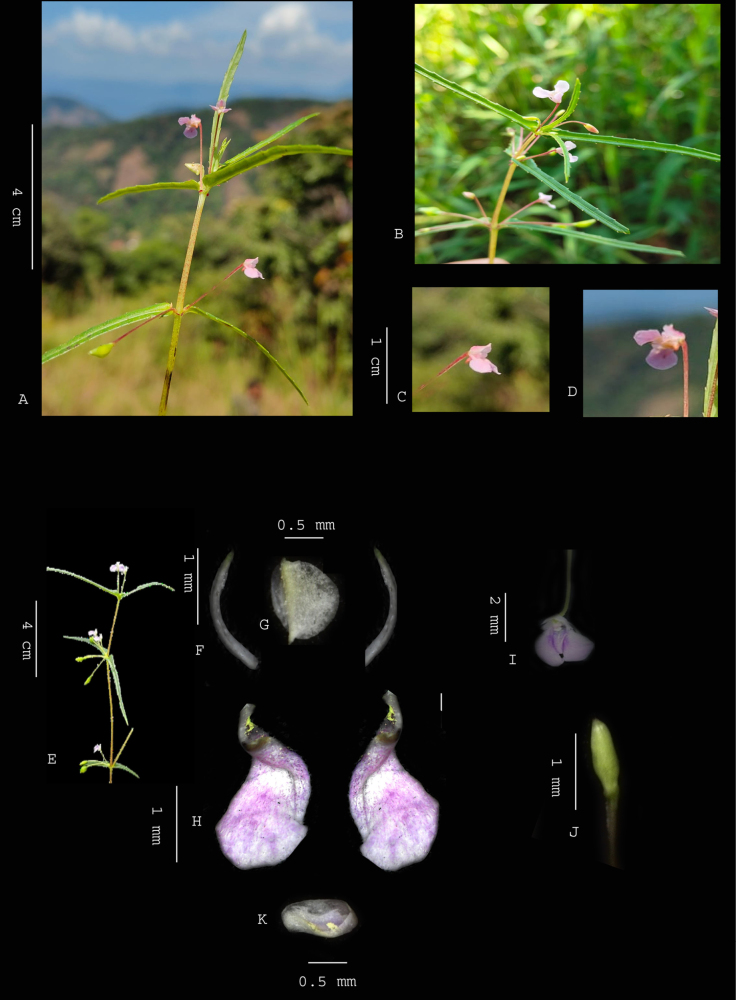
*Impatiens filcyii*. **A**. Habit; **B**. Single plant; **C**. Flower, side view; **D**. Flower showing spur; **E**. Single plant; **F**. Lateral sepal; **G**. Dorsal petal; **H**. Lateral united petal; **I**. Flower; **J**. Gynoecium; **K**. Lower sepal.

#### Diagnosis.

*Impatiens filcyii* is similar to *Impatiens periyarensis* B.Mani, Sinj.Thomas & Britto with respect to its upright habit, quadrangular stem, opposite decussate leaves and pink colored flowers but differs with respect to extrafloral nectaries (spathulate, toothed in *I. periyarensis vs*. absent in *I. filcyii*), inflorescence (4–5 flowers vs. 3–6 flowers), flowers 7–9 mm vs. 3–4 mm, shape of lateral sepal (falcate glabrous vs. linear-oblanceolate, pubescent), shape of dorsal petal (dorsally keeled, mucronate vs. not keeled entire), basal lobe (falcate vs. filiform), shape of distal lobe (obovate vs. broadly rectangular), dorsal auricle (prominent vs. absent), shape of lower sepal (funnel shaped vs. cucullate) and shape of capsule (fusiform vs. ovoid).

The species also shows similarity to *I. aliciae* Fisch. but differs with respect to the shape of lateral sepal (linear-oblong in *I. aliciae* vs. linear-oblanceolate in *I. filcyii*), basal lobe (falcate with red stripes vs. filiform without red stripes), distal lobe (obovate with a splash of purple-crimson streaks vs. broadly rectangular with absence of purple-crimson streaks), dorsal auricle (present vs. absent), shape of lower sepal (cymbiform vs. cucullate), and size of spur (3 mm, tubular-saccate vs. less than 1 mm, minute).

#### Description (macromorphology).

Annual, erect herb, 18–22 cm high. ***Stem*** simple to moderately branched, glabrous to sparsely pubescent, often slightly pubescent terete, with few scattered, brown, sessile or stipitate glands, particularly in the lower part of the stem, extrafloral nectaries absent. ***Leaves*** opposite, petiolate to subsessile; petiole up to 1.2 mm long; lamina ovate to oblanceolate or elliptic-lanceolate, 3.8–6.5 × 0.6–1.5 cm, base rounded with auricled lobes, apex acuminate, margins crenate, dentate to serrate or serrulate (usually in upper leaves) with cuspidate teeth; surfaces glabrous. Upper leaves alternate, sessile, oblong-lanceolate, smaller than lower leaves, apex acuminate, surfaces glabrous or sometimes with few glands, particularly on lower surface. ***Inflorescence*** peduncled, 3–6 flowered racemes arising from the axis of alternate leaves in the upper part of the stem purple, peduncle up to 4–4.2 cm long, glabrous, with small, brown spots, bracteoles absent, pedicels slender, 2–2.5 × 0.7–1.2 cm, glabrous, with or without sparse, brown spots. ***Flowers*** pink-purple; with yellow-purple blotch at throat 3–4 mm. ***Lateral sepals*** two, opposite, one on either side, falcate-oblanceolate, 0.6–1.2 × 0.7–0.9 mm, base convex, equally parted, glabrous, margins entire, apex acute, yellow tip, surface with faint green dots. ***Dorsal petal*** orbicular to oblong, 1.8–2.5 × 1.5–2 mm, apex slightly cordate, margins wavy, concave in the middle, winged on either side, not keeled from base to apex, 0.4–0.8 cm long. ***Lateral petals*** two lobed, basal lobe unequal, filiform, obtuse apex 3–4 × 3.2–4.2 mm, margins entire or wavy, distal lobe broadly rectangular with obtuse apex 6.2–7.3 mm, dorsal auricle absent. ***Lower sepal*** cucullate, white with a minute spur, 0.8 mm. ***Stamens*** 1–1.5 mm; filaments 0.3–0.5 mm long, anthers 0.8–2 mm long, partly fused. ***Gynoecium*** elliptical 8 mm. ***Ovary*** oblong-elliptic, 2–3 mm long, glabrous; style 0.1–0.4 mm long. ***Capsules*** ovoid, 1.8–2.3 cm long, glabrous, green with pink base and apex, 6–8 seeded. ***Seeds*** brownish-black, oblong or sub ovoid, 1.8–2.3 × 1.2–2.1 mm.

#### Description (micromorphology).

***Pollen*** two colpate, rod-shaped, 27.3–28.1 × 10.7–10.9 µm. Surface pattern highly reticulate forming an angular apocolpia which is prominent. Mesocolpia is faintly distinguished. Muri measures ca. 1–1.5 µm, baculate, lumina ca. 3.8 µm. intra and inter-luminar bacules are present, 12–13 in each, often free (Fig. 7).

#### Phenology.

August to November.

#### Etymology.

The specific epithet is named in honor of Prof. Filcy T. Baby, the former Head of the Department of Botany, CMS College Kottayam (Autonomous), Kerala, who was an admirable and outstanding teacher in the field of classical taxonomy. The epithet also expresses the corresponding author’s (TPR) gratitude and deep appreciation toward him as a mentor who fostered academic and scientific temper among students.

#### Habitat and distribution.

*Impatiens filcyii* is known only from the type locality, where it grows along temporary streams formed during the rainy season and along water-filled rock crevices. It is seen associated with *Cynoglossum zeylanicum* (Sw. ex Lehm.) Thunb. ex Brand, *Isachne kunthiana* (Wight & Arn. ex Steud.) Miq. and *Drosera indica* L.

#### Conservation status.

The new species is known from two subpopulations in the rocky terrain of Mamalakkandam, each separated by a distance of 250–500 m. The estimated extent of occurrence (EOO) is 2 km^2^, and the area of occupancy is less than 800 m^2^. The number of mature individuals in the area is expected to be 80–120. Since the population is narrow and prone to decline due to anthropogenic activity, further study of the population is preferred before assigning any conservation status. Therefore, it is recommended that the species be categorized as Data Deficient (DD) based on IUCN (2024).

#### Other notes.

*Impatiens filcyii* grows in open, muddy grasslands in the interior of Mamalakkandam. The habitat is similar to that of other *Impatiens* spp. found in this area. Yu et al. (2015) gave a worldwide classification of *Impatiens* into two subgenera, subgen. *Clavicarpa* Warb. and subgen. *Impatiens*. *Impatiens filcyii* belongs to sect. *Uniflorae* under subgen. *Impatiens*, characterized by capsules that are short-fusiform, conspicuously turgid in the middle region, ca. 1 cm long, an inflorescence that is a raceme with 1(–2) flowers, and ellipsoid seeds (Table 2). According to the classification by Bhaskar and Sringeswara (2017), *I. filcyii* belongs to sect. *Annuae*, characterized by opposite and decussate leaves and black-colored seeds.

**Table 1.** Morphological comparison between *I. flavispicata*, *I. courtallensis*, *I. herbicola*, *I. achudanandanii*, and *I. berchmansiensis*.

Characters	*I. flavispicata*	*I. courtallensis*	*I. herbicola*	*I. achudanandanii*	*I. berchmansiensis*
Habit	Erect herb	Erect-prostrate herb	Erect herb	Straggling herb	Erect-suberect herbs
Leaf	Linear to lanceolate, glabrous	Lanceolate-subcordate at base pubescent above, glabrous below	Linear truncate base, glabrous	Linear base truncate-ordate, sparsely pubescent	Lanceolate-obovate
Flower	1–2 flowers, white with yellow spot on the throat	Two per inflorescence, milky white	2–3 per inflorescence, white, bluish or yellowish	2–3 per inflorescence, creamy or whitish	One flower, yellow
Lateral sepal	0.5–1 mm long, linear-falcate, not nerved	3–5 mm linear ensiform inwardly curved prominently nerved	4–5 mm linear acuminate, not prominently nerved	Linear lanceolate aristate faintly nerved	Falcate, glabrous
Lower sepal	Cymbiform, not curved	Boat-shaped tip outwardly curved	Scapiformis, lanceolate	Boat-shaped, tip pointed	Pyriform
Dorsal petal	Ovate, beaked, dorsally thin keeled	Orbicular recurved thickly keeled apiculate	Orbicular, thickly keeled apiculate	Ovate, beaked, dorsally keeled	Ovoid-circular
Lateral united petals	Basal lobe minute digitate, distal lobe small obdeltoid	Basal lobe oblong, distal lobe round or spherical	Basal lobe oblong, distal lobe spherical	Basal lobe ovate, distal lobes ovate	Basal lobe roughly conical, distal lobe hatcher shape
Capsule	1–2 seeded, ovoid	3–5 seeded, fusiform	Gibbously ovoid, many seeded	Ovoid turgid, 2–6 seeded	2–3 seeded, lanceolate fusiform
Pollen	8–10 µm, ovoid, three colpate, white	16–18 µm, squarish four colpate, milky white	21–23 µm, radical 4–5 colpate, pale yellow	10–11 µm, whitish, two colpate	19.2–19.6 µm, four colpate, white pinkish

**Table 2.** Morphological comparison between *I. filcyii*, *I. ninanii*, *I. periyarensis*, *I. aliciae*, *I. cheruthoniensis*, and *I. josephia*.

Characters	*I. filcyii*	*I. ninanii*	*I. periyarensis*	*I. aliciae*	*I. cheruthoniensis*	*I. josephia*
Habit	Erect herb	Erect herb	Upright herb	Procumbent herbs	Erect herb	Erect herb
Leaf	Ovate-oblanceolate	Linear-lanceolate to ovate elliptical	Lanceolate	Narrowly elliptic oblong	Linear lanceolate	Linear
Flower	3–6 flowers, purple	4–5 flowers, purple	4–5 flowered white-pink	1–2 flowers deep pink or white	One flowered	3–5 flower pink
Lateral sepal	Falcate-oblanceolate, glabrous	Oblanceolate-oblong, sparsely pubescent	Falcate, glabrous	Lineate acute glabrous	Linear-lanceolate to aristate	Falcate mucronate puberulent
Lower sepal	Cucullate, no blotch	Navicular	Funnel-shaped apex forms a flap no blotch	Cymbiform sub acute blotch absent	Tubular	Boat-shaped apex cuspidate with orange blotch in the center
Dorsal petal	Orbicular-oblong	Orbicular to obovoid	Widely ovate	Orbicular	Ovate	Orbicular
Distal lobe	Broadly rectangular, asymmetrical	Ovoid- pyriform	Obovate asymmetrical	Broadly obovate, asymmetrical	Widely obovate	Orbicular apex retuse
Capsule	Ovoid	Ellipsoid	Fusiform	Ellipsoidal	Ellipsoidal	Fusiform asymmetrical
Pollen	27.3–28.1 µm, two colpate	13.6–1.1 µm, three colpate	Yellow (data not available)	Four colpate, white bilateral	Two colpate, pink, bilateral	Four colpate, white rod-shaped

#### Specimen examined (paratype).

*Impatiens filcyii* India. Ernakulam district, Koinipara, 1020 m a.s.l., 10.0988°N, 76.8166°E, 21 August 2024, Sindhu Arya, Salvy, Manuel, & Rogimon 3051 (RHK!).

### Impatiens ninanii

Taxon classificationPlantaeEricalesBalsaminaceae

Sindhu Arya, Salvy, Manuel & Rogimon sp. nov.

AEA3B29A-3C2A-5C4B-AD9E-4436132E320D

urn:lsid:ipni.org:names:77382026-1

Fig. 3

#### Type.

India. Kerala, Idukki district, Viripara, Mankulam, 10.0968, 76.8154, 980 m a.s.l., 29 October 2023, *Sindhu Arya, Rogimon, Salvy & Manuel, 3065* (holotype CMS!, isotype RHK!).

**Figure 3.**
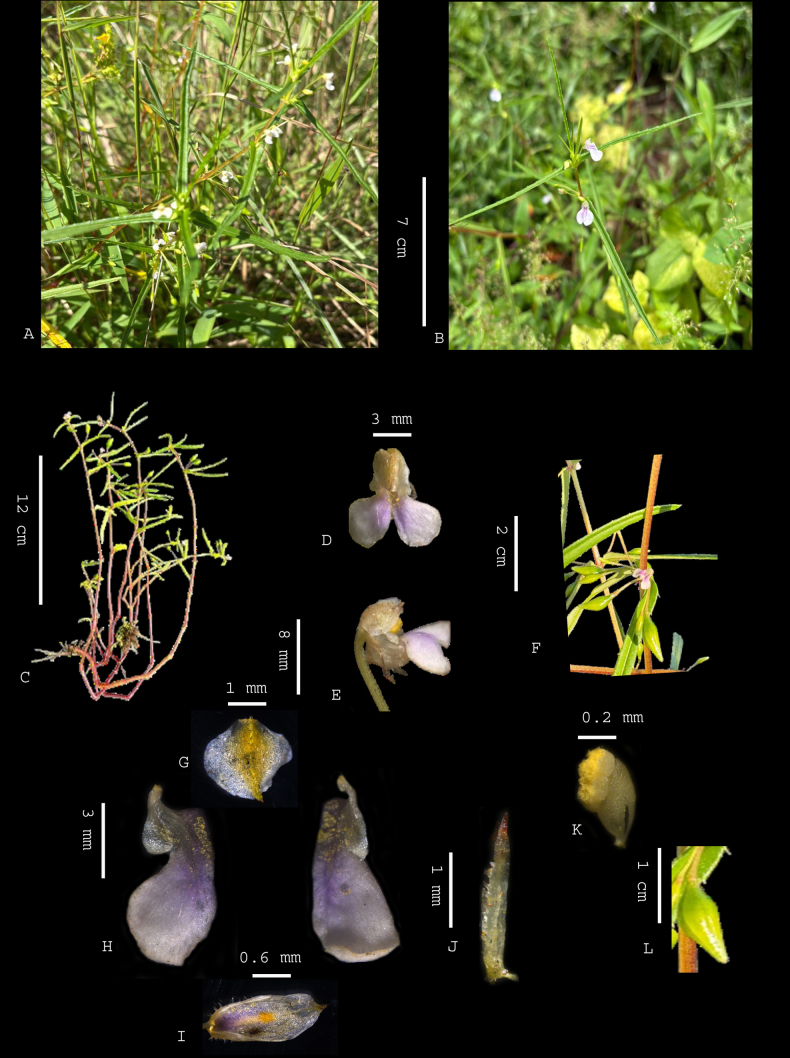
*Impatiens ninanii*. **A**. Habit; **B, C**. Single plant; **D**. Flower, front view; **E**. Flower, side view; **F**. Single flower; **G**. Dorsal petal; **H**. Lateral united petal; **I**. Lower sepal; **J**. Lateral sepal; **K**. Stamen; **L**. Fruit.

#### Diagnosis.

*I. ninanii* is similar to *I. cheruthoniensis* V.S.A.Kumar & Sindhu Arya in having succulent erect, glabrous habit differs with respect to leaf margin (serrate in *I. cheruthoniensis* vs. entire in *I. ninanii*), inflorescence (one or rarely two together vs. 4–5 flowers), lateral sepals (linear, lanceolate with aristate apex vs. oblanceolate-oblong with acute apex), lower sepal (tip pointed vs. tip curved), distal lobe (widely obovate vs. ovoid pyriform), basal lobe (conical vs. rectangular minute), dorsal auricle (absent vs. present).

#### Description (macromorphology).

Terrestrial erect herbs, 14–28 cm high. ***Stem*** cylindrical; faint purple dots throughout the stem. ***Leaves*** linear-lanceolate to ovate-elliptical, apex round or ovate, base conical, 6.2–6.4 × 2.3–3.4 cm, margin entire, sparsely pubescent; sessile to petiolate, petioles; up to 0.5 cm long, greenish brown. ***Inflorescence*** racemose, straight, 4–5 flowered 8–10 mm long, glabrous. ***Flowers*** clustered at the apex, purple, each 5–8 mm across; pedicels 0.8–1.0 cm long, bracts absent. ***Lateral sepals*** two, each 2.1–2.4 × 1.2–1.8 mm, oblanceolate-oblong, sparsely pubescent apex acicular. ***Lower sepals*** navicular curved at both the ends, spurred, spur minute slender, 1.0–1.2 mm long, milky white or straw color, straight. ***Dorsal petal*** broadly orbicular to obovoid, 2.3–2.4 × 3.2–3.4 mm; adaxially keeled, glabrous dull yellow or pale purple; keel mucronate, mucro ca. 1 mm long, pale green. ***Lateral united petals*** 2-lobed, violet to blue, basal lobe rectangular minute extends to an uplifted region; striations in violet color; distal lobes 0.5 cm long ovoid-pyriform broad rounded in the middle, dorsal auricle present ca. 2 mm. ***Stamens*** five, connate, ca. 1.5 × 1.6 mm; filaments white, anthers white. ***Ovary*** green, 1.8–2.1 × 1.0–1.2 mm, elliptic, broadly acute at apex, pubescent. ***Capsule*** pubescent, reddish green, broadly ellipsoid, apex acute, 1.2–1.3 cm long. ***Seeds*** 1–2, 0.8–1 mm long, glabrous.

#### Description (micromorphology).

***Pollen*** three colpate, cylindrical, 13.6–14.1 µm × 15.7–16.1 µm. Surface pattern faintly reticulates forming an angular apocolpia which is prominent. Minute dot-like appendages are seen associated with the apocolpia. Mesocolpia is distinguished. Muri measures 0.9–2.5 µm, baculate, lumina 1.8 µm. intra- and inter-luminar bacules are absent (Fig. 7).

#### Phenology.

July to October.

#### Etymology.

The specific epithet ninanii honors Prof. (Dr.) C.A. Ninan, former Head of the Department of Botany and Dean of the Faculty of Science at the University of Kerala, in recognition of his significant contributions to the field of plant science research.

#### Habitat and distribution.

The new species is found along muddy slopes of the rocky terrain associated with hillocks in Mankulam. *I. ninanii* is seen growing lushly along temporary rain streams flowing along the hillocks. The associated species include *Murdannia spirata* (L.) Bruckn., *Ludwigia perennis* L., and *Osbeckia octandra* DC.

#### Conservation status.

The new species is known from three subpopulations in the muddy grasslands of Viripara. The estimated extent of occurrence (EOO) is less than 1 km^*2*^, and the area of occupancy is less than 500 m^*2*^. The number of mature individuals in the area is expected to be 50–110. The locality is disturbed due to anthropogenic activity, and there is cultivated land adjoining the forest; hence, the likelihood of a decline in the number of individuals due to overgrazing and mudslides is high. Thus, further study of the population is preferred before designating any conservation status. Therefore, it is recommended that the species be categorized as Data Deficient (DD) based on IUCN (2024).

#### Other notes.

*Impatiens ninanii* belongs to sect. *Uniflorae* under subgen. *Impatiens*, characterized by the ellipsoidal shape of seeds and capsules that are short and conspicuously turgid in the middle. The new species also resembles *I. cheruthoniensis*, a species reported from the Cheruthoni region of Idukki, Kerala.

#### Specimen examined (paratype).

*Impatiens ninanii* India. Kerala, Idukki district, Pambumkayam, 1210 m, 10.0962°N, 76.9329°E, 10 August 2025, Sindhu Arya, Salvy, Manuel, & Rogimon 3074 ( RHK!).

### Impatiens xanthopetala

Taxon classificationPlantaeEricalesBalsaminaceae

Sindhu Arya, Manuel, Salvy & Rogimon sp. nov.

93C9331D-9103-5839-9AD1-59E592F6BE70

urn:lsid:ipni.org:names:77382027-1

Fig. 4

#### Type.

India. Kerala, Idukki district, Pambanar, 9.5744, 77.0144, 950 m a.s.l., 31 August 2025, *Sindhu Arya, Manuel, Salvy & Rogimon, 4102* (holotype CMS!, isotype RHK).

**Figure 4.**
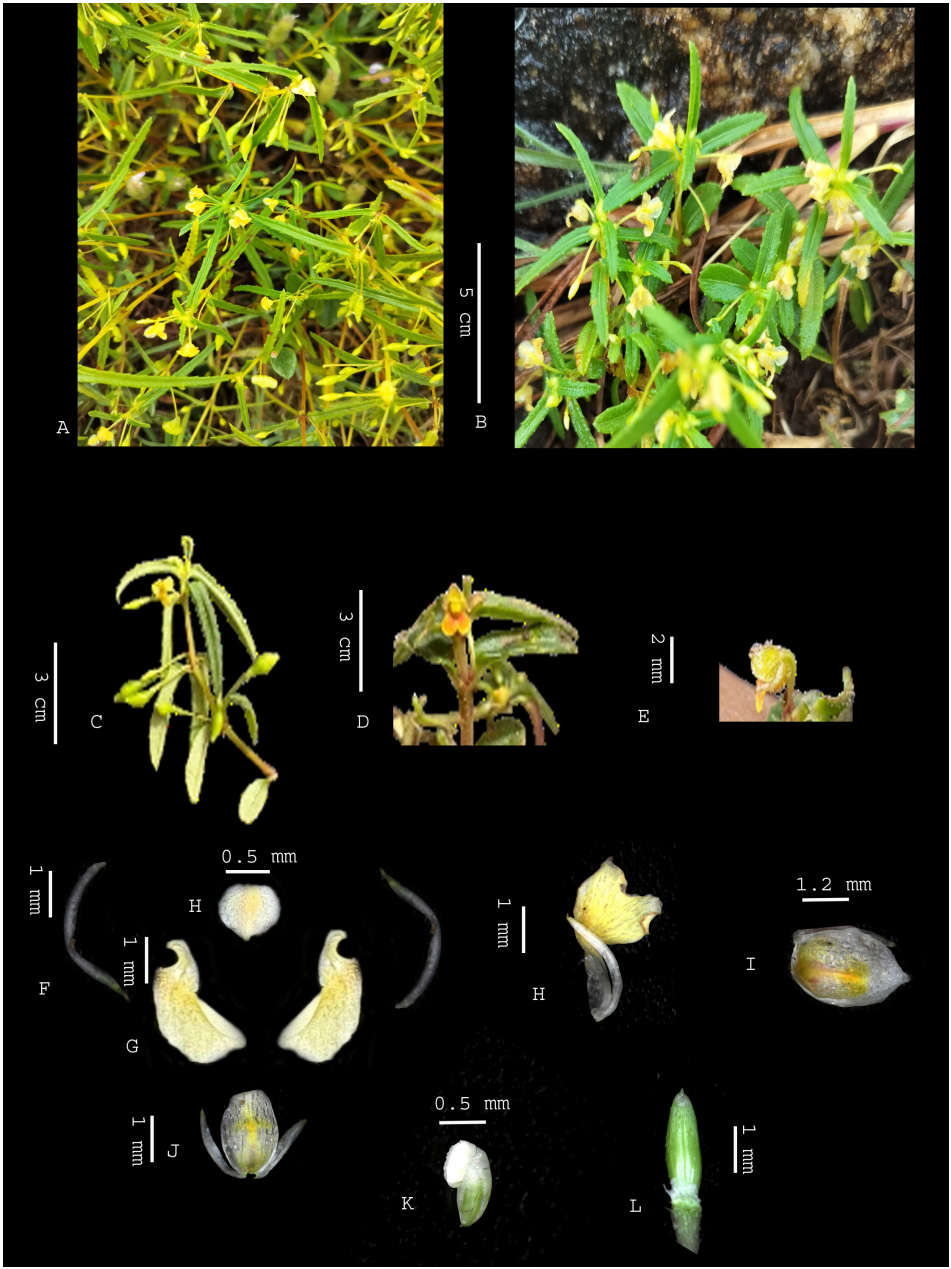
*Impatiens xanthopetala*. **A**. Habit; **B, C**. Single plant; **D**. Flower, front view; **E**. Flower, side view; **F**. Lateral sepal; **G**. Lateral united petal; **H**. Dorsal petal; **I**. Lower sepal showing blotch; **J**. Lower sepal; **K**. Stamen; **L**. Gynoecium.

#### Diagnosis.

*I. xanthopetala* shows similarity to *I. flavescens* with prostrate lateral branches that are pale greenish and opposite decussate leaves but differs with respect to extrafloral nectaries (two pairs of stipitate glands vs. absent), leaf blade (linear, elliptic vs. ovate-elliptic), dorsal petal (orbicular vs. narrowly ovoid), basal lobe (elliptic-oblong, not clawed vs. conical, deeply clawed), distal lobe (orbicular vs. dolabriform), lateral sepal (linear vs. falcate-lanceolate).

The species also shows similarity to *I. inconspicua* but differs with respect to petiole (sessile vs. 2 mm long), leaf base (linear, base round cuneate vs. ovate-elliptic, base mucronate), lateral sepal (linear subulate, 1-nerved vs. falcate-lanceolate 3-nerved), lateral petal (distal lobe-rhomboid, ovate vs. dolabriform), seeds (smooth *vs*. sparsely pubescent).

#### Description (macromorphology).

Annual, erect to straggling herb, succulent, often prostrate with many lateral branches 8–10 cm tall. ***Stem*** pale brown, terete, ridges absent, purple spotted branched towards the base, branches straggling, internodes elongate, nodes swollen, 1.5–3.5 cm long, extrafloral nectaries present. ***Leaves*** on lower part of the stem opposite, opposite to decussate towards apex of the stem, petiolate, petiole 2 mm, with extrafloral nectaries present downwards at the base of each leaf, leaf blade ovate to elliptic, 2.4–2.8 × 1.3–1.6 cm, base mucronate, sparsely pubescent, margin entire or serrate, apex acute to obtuse. ***Inflorescence*** raceme with 3–4 floral clusters at each node. ***Flowers*** 0.9–1.3 cm deep yellow with dark brown blotch on the throat, pedicellate, pedicel 1.2 cm long, filiform, terete with single row of hairs. ***Lateral sepal*** two, falcate to lanceolate, 0.8–1 mm long, subulate, nerve conspicuous, 3-nerved, yellow, pubescent, apex acuminate. ***Lower sepal*** conical, pubescent, apex acute, 0.4 to 0.8 × 0.2 to 0.3 mm, pale yellow, spur minute. ***Dorsal petal*** narrowly ovoid, 0.6 to 0.8 × 0.4 to 0.5 cm, prominently keeled, keel yellow uplifted, apex recurved acicular, glabrous. ***Lateral united petals*** two lobed, basal lobe 0.8 to 0.9 × 0.2–0.3 mm, conical, deeply clawed, small; distal lobe dolabriform without any spots 3–4 × 2–3 mm, tip obtuse without any streaks or spots. ***Gynoecium*** elongate to sub-cylindrical. ***Capsule*** fusiform, 1–1.3 cm long, 12–15 seeded, seeds ovoid, margin compressed, sparsely pubescent, black and shining.

#### Description (micromorphology).

***Pollen*** four colpate, roughly rectangular, 5.6 × 7.7 µm. Surface pattern reticulates forming a non-angular apocolpia which is prominent. Mamillae distributed throughout apocolpia. Mesocolpia is prominently distinguished. Muri narrow measures ca. 0.7–1.5 µm, baculate, lumina ca. 0.8 µm. intra- and inter-luminar bacules are present, 2–13 in each, often free or even fused (Fig. 7).

#### Phenology.

August to November.

#### Etymology.

The specific epithet xanthopetala corresponds to the striking prominent yellow color of the petal that helps easier recognition of the species in the field.

#### Habitat and distribution.

The new species *I. xanthopetala* is seen along shallow muddy areas associated with seasonal streams along the rocky cliff of Pambanar. The species is found to be associated with *Fimbristylis umbellaris* (Lam.) Vahl, *Emilia sonchifolia* (L.) DC., and *Exacum bicolor* Roxb.

#### Conservation status.

The new species is known from two subpopulations in a highly disturbed tourist spot, each separated by a distance of 5 km^2^. The estimated extent of occurrence (EOO) is 15 km^2^, and the area of occupancy (AOO) is less than 1 km^2^. The number of mature individuals is estimated to be 140–170 when considering all subpopulations. There is a possibility of this taxon occurring in nearby areas of Pambanar; therefore, it is recommended that the species be categorized as Data Deficient (DD) based on IUCN (2024).

#### Specimen examined (paratype).

*Impatiens xanthopetala* India. Idukki, Pattumala, 9.5792°N, 77.0344°E, 1175 m, a.s.l, 9 November 2025, Salvy & Rogimon 4119 (RHK!).

#### Taxonomic notes.

*Impatiens xanthopetala* belongs to sect. *Uniflorae* under subgen. *Impatiens*, characterized by the ellipsoidal shape of the capsule (Yu et al. 2015). The morphological differences of *I. xanthopetala* with *I. inconspicua* Benth. ex Wight & Arn. and *I. flavescens* Karupp. & V. Ravich. are listed in Table 3. The new species also shares slight similarity with *I. megamalayana* but differs in the shape of the lateral sepals (sickle-shaped in *I. megamalayana* Ramas.), lower sepal (wrinkled, sparsely hairy), and capsule (ovoid). A key to delineate the new species from its allied taxa is given below:

**Table 3.** Morphological comparison between *I. xanthopetala*, *I. flavescens*, *I. inconspicua*, and *I. megamalayana*.

Characters	*I. xanthopetala*	*I. flavescens*	*I. inconspicua*	*I. megamalayana*
Habit	Erect to straggling herb	Erect	Erect to sub-erect herb	Erect herb
Leaf	Ovate-elliptic	Linear-lanceolate	Linear	Ensiform
Flower	3–4 flowers in cluster, deep yellow with a dark brown blotch	2–3	3–4 pink or white	1–2 flowers, pale purple with deep reddish yellow spot
Lateral sepal	Falcate-lanceolate	Linear	Linear subulate	Sickle-shaped apex acuminate
Lower sepal	Conical	Smooth, falcate	Smooth, saccate	Saccate
Dorsal petal	Narrowly ovoid	Orbicular	Orbicular	Orbicular
Lateral united petals	Dolabriform	Orbicular	Rhomboid ovate	Oblong spherical
Fruit	Fusiform	Fusiform	Ovoid	Ovoid
Pollen	Four colpate	Three colpate	Two colpate	Three colpate

##### Key to the species

**Table:** 

1	Herbs reaching a height of 15 cm; lateral sepals with inconspicuous nerves	***I. flavescens***
–	Herbs reaching a height of up to 40 cm; lateral sepals 1–3 nerved	**2**
2	Lateral sepals sickle-shaped, 1–2 nerved; leaf blade ensiform	***I. megamalayana***
–	Lateral sepals linear or falcate, 1–3 nerved; leaf blade linear or ovate-obovate	**3**
3	Lateral petals with terminal lobe rhomboid-ovate; capsule ovoid	***I. inconspicua***
–	Lateral petals with terminal lobe dolabriform; capsule fusiform	***I. xanthopetala***

### Impatiens berchmansiensis

Taxon classificationPlantaeEricalesBalsaminaceae

Sindhu Arya, Salvy, Manuel & Rogimon sp. nov.

54D10A52-3377-5BD4-A87B-521FCFE84268

urn:lsid:ipni.org:names:77382028-1

Figs 5, 6

#### Type.

India. Kerala, Idukki district, Vagamon, along the muddy slopes of highland, 9.6886, 76.9069, 850 m a.s.l., 20 October 2024, *Sindhu Arya, Salvy, Manuel & Rogimon, 4128* (holotype CMS!, isotype RHK!).

**Figure 5.**
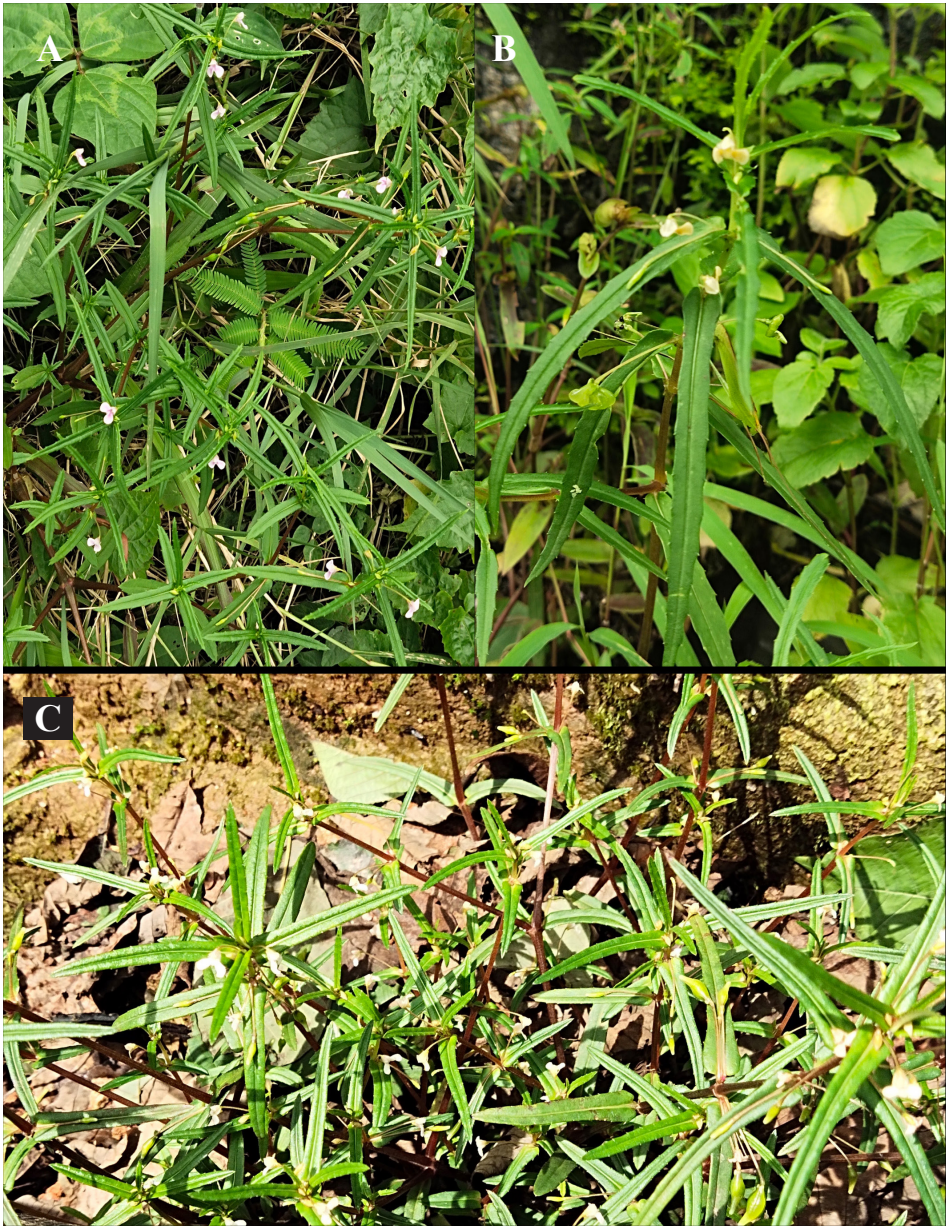
*Impatiens berchmansiensis*. **A, B**. Habit.

**Figure 6.**
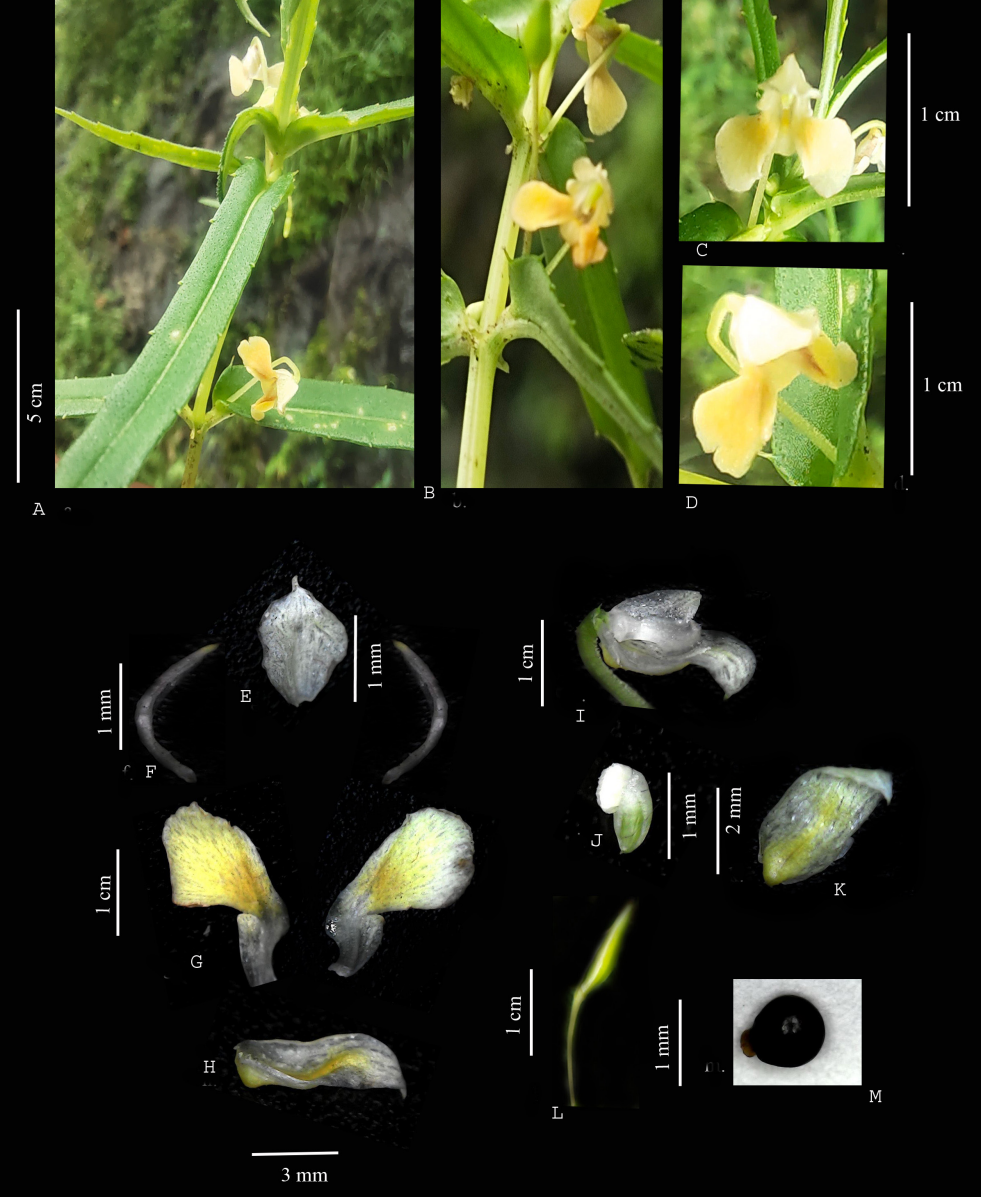
*Impatiens berchmansiensis*. **A, B**. Single plant; **C**. Flower, front view; **D**. Flower, side view; **E**. Dorsal petal; **F**. Lateral sepal; **G**. Lateral united petal; **H**. Lower petal; **I**. Flower; **J**. Stamen; **K**. Lower sepal showing blotch; **L**. Fruit; **M**. Seed.

#### Diagnosis.

*Impatiens berchmansiensis* is most similar to *I. herbicola* Hook.f. in overall habit and having linear leaves but differs by the color and size of the flowers (yellow 4–8 mm in *I. berchmansiensis* vs. white-purple 3 mm in *I. herbicola*), stem (quadrangular vs. terete), lateral sepal (falcate vs. linear), the shape of the dorsal petal (ovoid-circular vs. orbicular), dorsal auricle (prominent vs. absent) the shape of the lateral united petals (basal lobe conical, apex slightly acute; distal lobe hatcher-shaped vs. basal lobe oblong, distal lobe round or spherical), the shape of lower sepal (pyriform with apex acuminate and incurved vs. scaphiformis with apex straight), spur (present vs. absent), the shape of the fruit (fusiform vs. ovate) the shape, number and hairiness of the seeds (ovoid, 2–3 seeded and seed with minute hairs vs. fusiform, 5–8 seeded and glabrous) and the size and color of the pollen grains (19.2 × 18.7 μm white-pinkish in vs. 12 × 13 μm squarish yellow).

#### Description (macromorphology).

Annual herbs, erect to suberect, 14–16 cm tall. ***Stem*** simple unbranched, green with purple spots, quadrangular at base, pubescent on younger shoots, lower region glabrous, extrafloral nectaries absent. ***Leaves*** simple, opposite decussate towards the base of the stem, whorled towards the apex; lamina 2.3–3.3 × 0.8–1.0 cm, lanceolate-obovate, serrate at margins, cuneate at base, obtuse at apex, green to pale green, glabrous on both the surfaces. Petioles 1.2–1.3 cm long, grooved, hairy. ***Inflorescence*** single flowered raceme; peduncles 3–3.2 cm long, green to purplish brown; pedicels 1.9–2.1 cm long. ***Flowers*** 0.4–0.8 × 0.3–0.4 cm, yellow. ***Lateral sepal*** 2–2.2 × 0.3–0.4 mm, falcate, white, apex acute, yellow, glabrous. ***Lower sepal*** pyriform, entire at margins, acuminate at apex, recurved inwards, spur up to 1 mm long, straight. ***Petals*** yellow, delicate. ***Dorsal petal*** 1.1–1.8 × 1.1–1.4 mm, ovoid circular with apex acute and reflexed, entire at margins, rounded at base and apex, glabrous. ***Lateral united petals*** 1.5–1.7 × 0.6–0.7 mm, entire at margins, yellow; basal lobe roughly conical, apex acute, yellow; distal lobe hatcher-shaped, yellow, dorsal auricle present. ***Stamens*** five; filaments 0.8–0.9 mm long, united, white, papery; united, oval, extrorse, pink-white, longitudinally dehiscing. ***Ovary*** 0.8–1 × 1 mm, elliptic, pale green to yellow; style short; stigma sticky. ***Capsules*** 1.5–1.8 cm, short lanceolate-fusiform, acute at apex, green, 2–3 seeded. ***Seeds*** 0.8–0.9 mm, ovoid with minute hairs.

#### Description (micromorphology).

**Figure 7.**
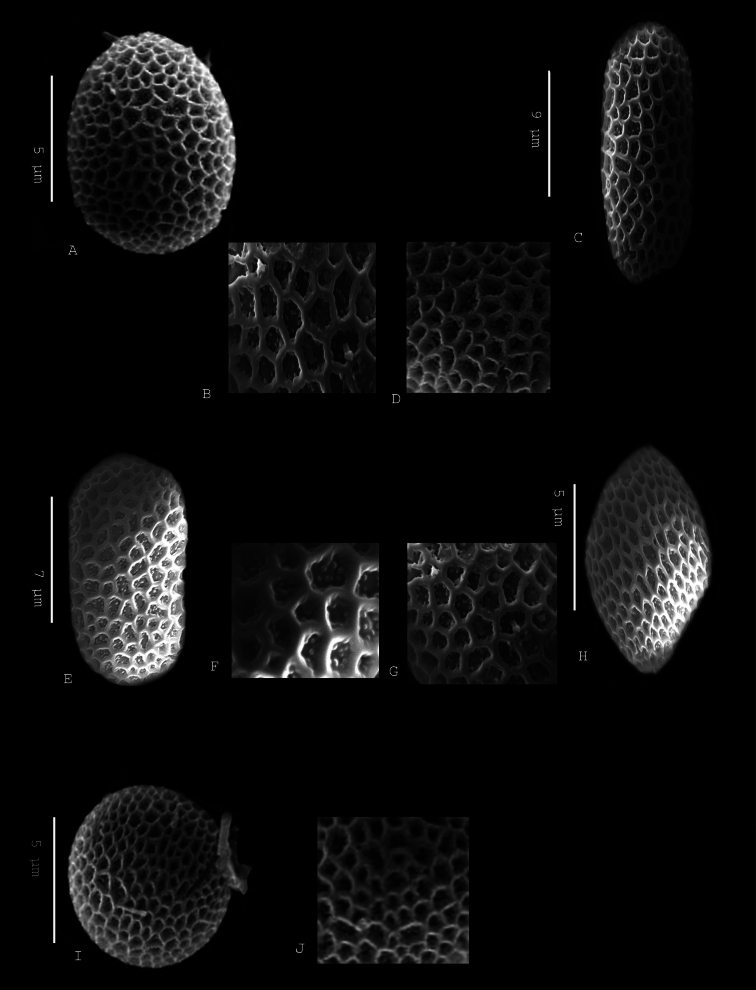
Micromorphology (pollen). **A**. *I. flavispicata*; **B**. Surface enlarged; **C**. *I. filcyii*; **D**. Surface enlarged; **E**. *I. ninanii*; **F**. Surface enlarged; **G**. *I. xanthopetala*, surface enlarged; **H**. *I. xanthopetala*; **I**. *I. berchmansiensis*; **J**. Surface enlarged.

***Pollen*** four colpate, ovoid, 19.2–19.7 × 18.7–18.9 µm. Surface pattern reticulate. Apocolpia densely reticulate with small pores on the muri. Mesocolpia is faintly distinguished. Muri measures ca. 0.5–0.9 µm, baculate, lumina ca. 0.8 µm. intra- and inter-luminar bacules are present, 9–10 in each, fused (Fig. 7).

**Figure 8.**
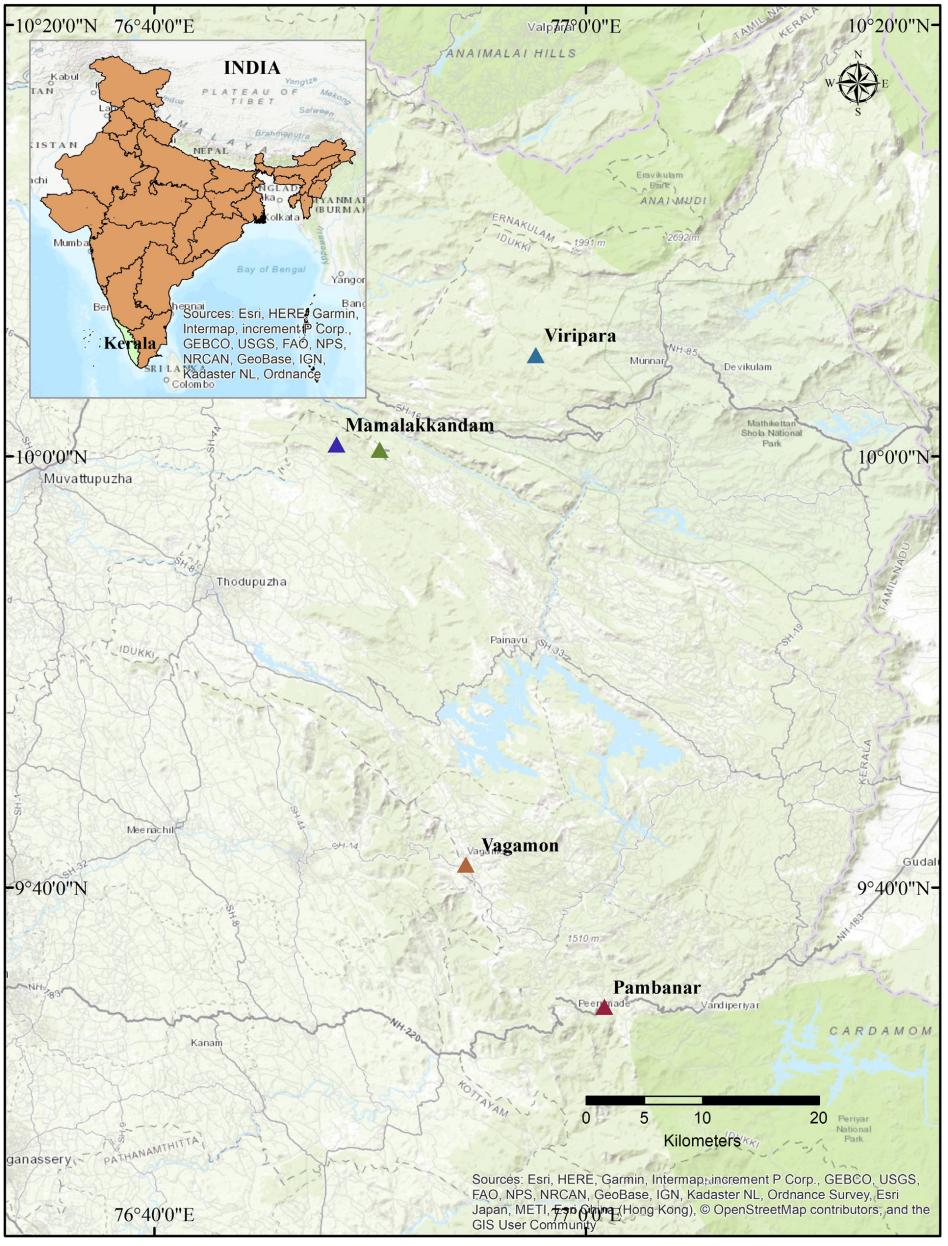
Distribution map of *I. flavispicata*, *I. filcyii*, *I. ninanii*, *I. xanthopetala*, and *I. berchmansiensis*.

#### Phenology.

August to November.

#### Etymology.

The specific epithet berchmansiensis was chosen in honor of St. Berchmans College (Autonomous), Changanassery, Kerala, the alma mater of the second author (TS), in recognition of its incessant contributions to education, research, and the advancement of plant sciences. The college was founded in 1922 and is a pioneer and leading higher educational institution in India. The name also reflects the author’s gratitude and affection toward the institution that nurtured their academic and scientific pursuits.

#### Habitat and distribution.

The new species *I. berchmansiensis* is seen along muddy slopes at an altitude of 800–1,100 m a.s.l. The species is found to be associated with *Utricularia caerulea* L., *Drosera indica* L., and *Rhynchoglossum notonianum* (Wall.) B.L.Burtt.

#### Conservation status.

The new species is known from different subpopulations in the Wagamon area, each separated by a distance of 2 km. The estimated extent of occurrence (EOO) is 10 km^2^, and the area of occupancy (AOO) is less than 1 km^2^ in the present study. The number of mature individuals is estimated to be 100–150 when considering all localities. The chances of occurrence of the new taxon in other localities of the Western Ghats cannot be neglected. Hence, it is preferred to assign *Impatiens berchmansiensis* to the Data Deficient (DD) category (IUCN 2024).

#### Taxonomic notes.

Yu et al. (2015) gave a worldwide classification of *Impatiens* into two subgenera and sections. *Impatiens berchmansiensis* belongs to sect. *Uniflorae* under subgen. *Impatiens*, characterized by an inflorescence that is a raceme with 1(–2) flowers and ellipsoid seeds. The morphological differences of *I. berchmansiensis* with its allied taxon *I. herbicola* are summarized in Table 1. Color variation among the flowers of *Impatiens herbicola* is common within the population, but in *I. berchmansiensis*, no such observations were noted. Furthermore, the pollen color is white to pinkish in *I. berchmansiensis*, whereas it is yellow in *I. herbicola*. The consistency was further confirmed by the shape and size of the pollen. In *I. herbicola*, the pollen grains are squarish or radial (Bhaskar 2012), and the sexine is reticulate, the lumen is wider, and the muri are thin toward the periphery (Mani and Thomas 2017). The present observation on *I. berchmansiensis* revealed rod-shaped pollen that is larger, with narrow lumen and muri uniformly thick on all sides. The species shows similarity to *I. brittoi* B.Mani & S.Thomas in terms of pollen having thick muri and narrow lumen, but the pollen is rod-shaped in *I. berchmansiensis* vs. ellipsoidal in *I. brittoi*. In addition, the salmon-red stipules and pedicels, axillary 3–5-flowered fascicles, navicular lip with a prominent orange blotch, 0.5 mm-long dorsal auricle, and fusiform capsules mentioned as the diagnostic features of *I. brittoi* are completely absent in *I. berchmansiensis*.

#### Specimens examined (paratype).

*Impatiens berchmansiensis* India. Idukki district, Kuttikkanam, 9.5780°N, 76.9720°E, 1050 m. a.s.l, 24 November 2024, Salvy & Rogimon 4137 (RHK!).

## Supplementary Material

XML Treatment for Impatiens flavispicata

XML Treatment for Impatiens filcyii

XML Treatment for Impatiens ninanii

XML Treatment for Impatiens xanthopetala

XML Treatment for Impatiens berchmansiensis

## Additional information

### Artificial Intelligence (AI) use

The authors accept full responsibility for the content of the manuscript, including the disclosure of any use of AI.

No AI tools were used in the preparation of this manuscript.

### Author ORCIDs

S. Arya https://orcid.org/0000-0002-2029-2456

T. Salvy https://orcid.org/0009-0008-9054-4533

T. Manuel https://orcid.org/0000-0002-0782-5465

T.P. Rogimon https://orcid.org/0000-0001-8364-1679

### Data availability

All of the data that support the findings of this study are available in the main text.
