# Corrigendum: Arya S, Salvy T, Manuel T, Rogimon TP (2026) Five new species of *Impatiens* (Balsaminaceae) from southern Western Ghats, Kerala. PhytoKeys 276: 275–295. https://doi.org/10.3897/phytokeys.276.200226

**DOI:** 10.3897/phytokeys.279.207696

**Published:** 2026-09-30

**Authors:** Sindhu Arya, Thomas Salvy, Thomas Manuel, Thomas Plammoottil Rogimon

**Affiliations:** 1 Department of Botany, St Berchmans College (Autonomous), Changanassery, Kerala - 686101, India Department of Botany, St Berchmans College (Autonomous) Changanassery India; 2 Karimpanackel House, Neeloor P.O., Kottayam, Kerala - 686651, India Karimpanackel House, Neeloor P.O. Kottayam India; 3 Department of Botany, CMS College Kottayam (Autonomous), Kottayam, Kerala - 686001, India Department of Botany, CMS College Kottayam (Autonomous) Kottayam India

In our recent work (Arya et al. 2026) several errors in the manuscript have been identified and brought to our attention. These errors do not affect the overall taxonomic conclusions, diagnosis, descriptions, nomenclatural or general findings of the study. Corrections to the manuscript are provided below.

1. Correction of geographic coordinates.

The holotype coordinates provided for *Impatiens filcyii* and *Impatiens ninanii* were reported incorrectly during manuscript preparation. As a result, identical coordinates appeared for the two species despite their occurrence in different localities.

The correct holotype locality information is as follows:

*Impatiens filcyii*: Mamalakkandam, Ernakulam District, Kerala, India – (10.0968, 76.8154).

*Impatiens ninanii*: Viripara, Mankulam, Idukki District, Kerala, India – (10.0781, 76.9615).

The distribution map was prepared using the correct field localities; therefore, the correction of coordinates does not alter the distribution of either species.

2. Correction of pollen size for *Impatiens herbicola*.

An inconsistency occurred in the reported pollen dimensions of *Impatiens herbicola*. The value reported on page 288 was incorrect due to a typographical error. The correct pollen size is: 21 × 23 µm.

3. In the comparative account of *Impatiens achudanandanii*, certain characters were reported inaccurately. The correct information follows the original description published by (Arya et al. 2021).

The species should be treated as a glabrous herb and its pollen grains measure 10 × 16 µm as reported in the protologue.

4. On page 279 under the description of *Impatiens filcyii* the inflorescence is given as “3–6 flowered racemes arising from the axis of alternate leaves.” Here the word alternate was an error and it is to be read as “3–6 flowered racemes arising from the axis of leaves.” Phyllotaxy is opposite as mentioned in the description.

5. In the Introduction the updated number of *Impatiens* from India was not provided, so it may be read as:

“In India the genus *Impatiens* is represented by 345 taxa (Prabhukumar et al. 2022).”

6. On page 278 under the section habitat and distribution of *I. flavispicata*, it states that “each plant spreads over an area of 5–10 m^2^.” It is to be corrected and should read as “each population is spread over an area of 5–10 m^2^.”

The authors regret these mistakes and apologize for any inconvenience caused to readers.

## Additional information

### Artificial Intelligence (AI) use

The authors accept full responsibility for the content of the manuscript, including the disclosure of any use of AI.

No AI tools were used in the preparation of this manuscript.

### Author ORCIDs

S. Arya https://orcid.org/0000-0002-2029-2456

T. Salvy https://orcid.org/0009-0008-9054-4533

T. Manuel https://orcid.org/0000-0002-0782-5465

T.P. Rogimon https://orcid.org/0000-0001-8364-1679

### Data availability

All of the data that support the findings of this study are available in the main text.
